# Emerging Applications of Metabolomics in Traditional Chinese Medicine Treating Hypertension: Biomarkers, Pathways and More

**DOI:** 10.3389/fphar.2019.00158

**Published:** 2019-03-08

**Authors:** Mingxiao Yang, Lixing Lao

**Affiliations:** ^1^School of Chinese Medicine, LKS Faculty of Medicine, The University of Hong Kong, Hong Kong, China; ^2^Department of Chinese Medicine, The University of Hong Kong-Shenzhen Hospital, Shenzhen, China

**Keywords:** traditional Chinese medicine, metabolomics, hypertension, metabolic regulation, metabolome

## Abstract

Hypertension is a prevalent, complex, and polygenic cardiovascular disease, which is associated with increased mortality and morbidity. Across the world, traditional Chinese medicine (TCM) constituted by herbal medicine and non-pharmacological therapies is used to assist blood pressure management. Though widely accepted in daily practice, its mechanism remains largely unknown. Recent years saw a number of studies utilizing metabolomics technologies to elucidate the biological foundation of the antihypertensive effect of TCM. Metabolomics is a relatively “young” omics approach that has gained enormous attention recently in cardiovascular drug discovery and pharmacology studies of natural products. In this review, we described the use of metabolomics in deciphering TCM diagnostic codes for hypertension and in revealing molecular events that drive the antihypertensive effect. By corroborating the diagnostic rules, there's accumulating evidence showing that metabolic profile could be the signature of different syndromes/patterns of hypertension, which offers new perspectives for disease diagnosis and efficacy optimization. Moreover, TCM treatment significantly altered the metabolic perturbations associated with hypertension, which could be a crucial mechanism of the therapeutic effect of TCM. Not only significantly rebalances the dynamics of metabolic flux, TCM but also elicits metabolic network reorganization through restoring the functions of key metabolites, and metabolic pathways. The role of TCM in regulating metabolic perturbations will be informative to researchers seeking new leads for drug discovery. This review further envisioned the promises of employing metabolomics to explore network pharmacology, host-gut microbiota interactions and metabolic reprogramming in TCM, and possible herb-drug interactions in this field in future.

## Introduction

Hypertension is a prevalent cardiovascular disease that leads to increased morbidity and mortality (Forouzanfar et al., 2017). It is the major cause of many irreversible cardiovascular events such as stroke, chronic heart failure, and cerebrovascular death (Lloyd-Jones et al., 2010). Another consequence is target organ damage such as dementia and chronic kidney diseases (Faraco and Iadecola, 2013; Webster et al., 2017). Due to the intricate pattern of the pathology, hypertension requires systematic management program to dismiss both genetic and environmental pathogenic factors. As the standard care, a plethora of antihypertensive drugs is accessible for patients, while the reality of blood pressure control is suboptimal. In fact, despite therapeutic effects, drug therapy is associated with side effects and increased risk of cardiovascular events (Dunder et al., 2003; Brouwers et al., 2014); patient's low compliance to medications also disadvantages the benefits of drugs (Krousel-Wood et al., 2004).

As a result, present blood pressure management paradigm pays increasing attention to non-pharmacological therapies. Contemporary clinical guidelines have placed alternatives interventions in an equally critical position as pharmacotherapies and advocate the use of non-pharmacological interventions at least since the prehypertension stage (130–140 mmHg/80–90 mmHg). The American College of Cardiology/American Heart Association recommends patients to adopt non-pharmacological interventions, such as weight management, healthy diet with reduced intake of dietary sodium, and active physical exercise in the early stage of hypertension (Whelton et al., 2018). Moreover, in many countries of the world phytomedicine and alternative medicine are frequently consumed by patients to enhance the therapeutic effect of conventional drugs. People who use traditional Chinese herbal medicine (TCM) represent a substantial proportion of this population. Though a growing body of evidence demonstrated the potentials of traditional Chinese medicine as an effective treatment for hypertension, more studies are warranted (Flachskampf et al., 2007; Xiong et al., 2012; Wang et al., 2013a).

Recent years saw a large majority of studies utilizing metabolomics technologies to elucidate the biological foundation of the antihypertensive effect of TCM. In this review, we describe the use of metabolomics in explaining the principles of TCM syndrome differentiation for hypertension, in revealing the mechanism of the antihypertensive effect of TCM, and the potentials of utilizing metabolomics to deal with questions existed in TCM treating hypertension. This review also offers perspectives for future metabolomics studies in using TCM, and, at large, natural products or alternative therapies, to treat hypertension.

## Systems Biology in Hypertension Research and the Logic of Metabolomics

The disease for individual is regarded as the consequence of a compilation of molecular and cellular events predominated by genetic predispositions and environmental exposures. To probe the complex pathology requires system-level analytical methodologies that possess the power to precisely capture the holistic physiological reactions in the whole process. System biology approaches that integrate multi-omics technologies and computing sciences are necessary tools to realize this goal (Hood and Friend, 2011; Chen and Snyder, 2013). Unlike genomics and other omics easy to be confounded by post-transcriptional or post-translational changes and epigenetics, metabolomics is directly connected with disease phenotype (Fiehn et al., 2000). It is able to offer insights into metabolic phenotype and the correlations between metabolic perturbations and disease. Metabolomics approach identifies “all” small molecules (<1,500 Da) within biological samples (biofluids, such as urine, cerebrospinal fluid, saliva, serum, plasma in human, and other tissues) (Wishart, 2007a). Those small molecules, known as metabolites, are categorized as endogenous metabolites and exogenous metabolites according to their biological sources, and both are essential constituents of the human metabolome (Dunn et al., 2012). Biologically, endogenous metabolites are the final products of gene and protein activities. As its number is fewer than genes, transcripts and proteins, there is less data available to be interpreted as compared to other omics (Karahalil, 2016).

While, exogenous metabolites are synthesized, metabolized and excreted by the human intestinal microbial flora (Wishart, 2008). However, due to the considerable variations in the gut microbial species and environmental exposures, human metabolome is significantly varied in individual levels. At present, no single instrument or analytical chemistry method can fulfill the mission of positive identification of the entity of the human metabolome. The tandem use of multiple analytical instrumentations is able to provide system-level putative identification of metabolites (Patti et al., 2012). Nevertheless, the current analytical methods of metabolomics are still limited in terms of sensitivity, reproducibility, and consistency, depending on different platform/protocol used. Annotation of “unknowns” and validation of their biological properties may represent other challenges in metabolomics studies. In recent years, progressions such as databases construction, *in-silico* fragmentation and automatic annotation, have advanced the development of metabolomics in solving these problems.

Hypertension is a complex, polygenic disease. Both genetic dispositions and environmental exposures contribute to its onset and progression. Their interactions fluctuate the actions of enzymes that catalyze blood pressure regulation and result in perturbations of the human metabolome. Consequently, related metabolites as the “end-products” drive cardiovascular phenotypic changes (in this case, persistent escalation of blood pressure). To metabolomics approach, all metabolites are detectable and putatively characterizable. Therefore, it can be used to depict the metabolic aberrations occurred during the pathological process of hypertension. Furthermore, hypertension is an essential element of the metabolic syndrome, which has been confirmed by plenty of studies to be intercorrelated with disturbed substance metabolism, such as sugar and lipid metabolism. Employing metabolomics approach will further reveal new pathophysiology of hypertension and its associated target organ damages. With such tool, early detection and prediction of target organ damages become possible (Currie and Delles, 2017). This opens new windows for the recognition of disease pathology, the identification of new diagnostic biomarkers, and consequently the discovery of new therapeutic tools (Coen et al., 2008; Sreekumar et al., 2009). A detailed review of hypertension metabolomics is provided by Nikolic et al. (2014) and Arnett and Claas (2018).

## Metabolomics in TCM Syndrome Differentiation of Hypertension

TCM emphasizes personalized diagnosis, but the wisdom used in analyzing disease pattern is different from western medicine, which is mostly symptom-based. According to TCM theories, hypertension can be categorized into four syndromes, including Liver-Fire Ascending (LF), Yin Deficiency-Yang Hyperactivity (YD), Yin-Yang Deficiency (YY), and Dampness-Phlegm Accumulation syndromes (DP). Etiologically, TCM believes that LF syndrome is primarily caused by the deficiency of kidney yin, which subsequently loses its control over liver Yang. The exuberating *Yang* of the liver goes upward and causes symptoms such as dizziness and headache, etc. Many genetic factors, such as aging, genetic dispositions, and emotional factors are acclaimed to be responsible for this syndrome. Jiang et al. found that the urine and blood samples of LF presented salient abnormalities in amino acid and glucose metabolism as compared with healthy controls (Jiang et al., 2010, 2012b). By constructing a metabolic network, they further linked this syndrome to a disrupted metabolic network that features on abnormal noradrenaline, hexanoic acid, and arachidonic acid metabolisms (Jiang and Li, 2013).

However, due to the lack of valid comparators such as another TCM syndrome, one can only infer that metabolic perturbations exist in LF syndrome, but not able to “metabolically tag” this syndrome with such metabolic perturbation. Further subgroup analysis to directly compare the metabolic features of different TCM syndromes is necessary. Li et al. conducted a metabolomics analysis of plasma samples of YD patients, YY patients, and healthy subjects and found that each pattern has its unique pattern of metabolism (Li et al., 2013). YD patients have been related to uncoordinated sympathetic nervous system activation and YY associated with low metabolic rate. Furthermore, because Yin deficiency is recognized as a major pathology to both YD and YY syndrome, this study also found that abnormal glucose metabolism is common to both YD and YY, indicating the significant role of glucose metabolism in Yin deficiency. Another TCM pattern of hypertension is the accumulation of damp-phlegm caused by spleen and stomach deficiency, which connects blood pressure regulation to the digestive tract. Wu et al. found that elevated uric acid could be a biomarker of DP syndrome as compared with other syndromes and normotensive condition (WU T. et al., 2016). Moreover, the metabolic pattern of DP syndrome featured on the increase of citric acid, alanine, low-density lipoprotein (LDL), very low-density lipoprotein (VLDL), and the decrease of glucose, lysine, proline, lactose in blood when compared with LF.

Characterizing the biological basis of TCM syndrome of hypertension is the premise to understand the mechanism of TCM treatment for hypertension. There are insufficient research tools to portray the whole picture of TCM diagnosis in the early days. Metabolomics and other analytical technologies bridge this gap between research demands and tools available. Because metabolomics helps to unravel the constellation of TCM diagnosis, the hypertensive population can be further stratified for the optimization of therapeutic effect. The elucidation of the metabolic pattern for different TCM syndromes is also the root for understanding the actions of TCM antihypertensives. Network pharmacology stresses that the application of metabolomics and other omics technologies in TCM studies will yield “network target and multicomponent therapeutics,” which will potentiate cardiovascular drug discovery (Li et al., 2014).

## Metabolomics Revealing Mechanisms of Action of TCM Antihypertensive Therapies

**Figure 1:**
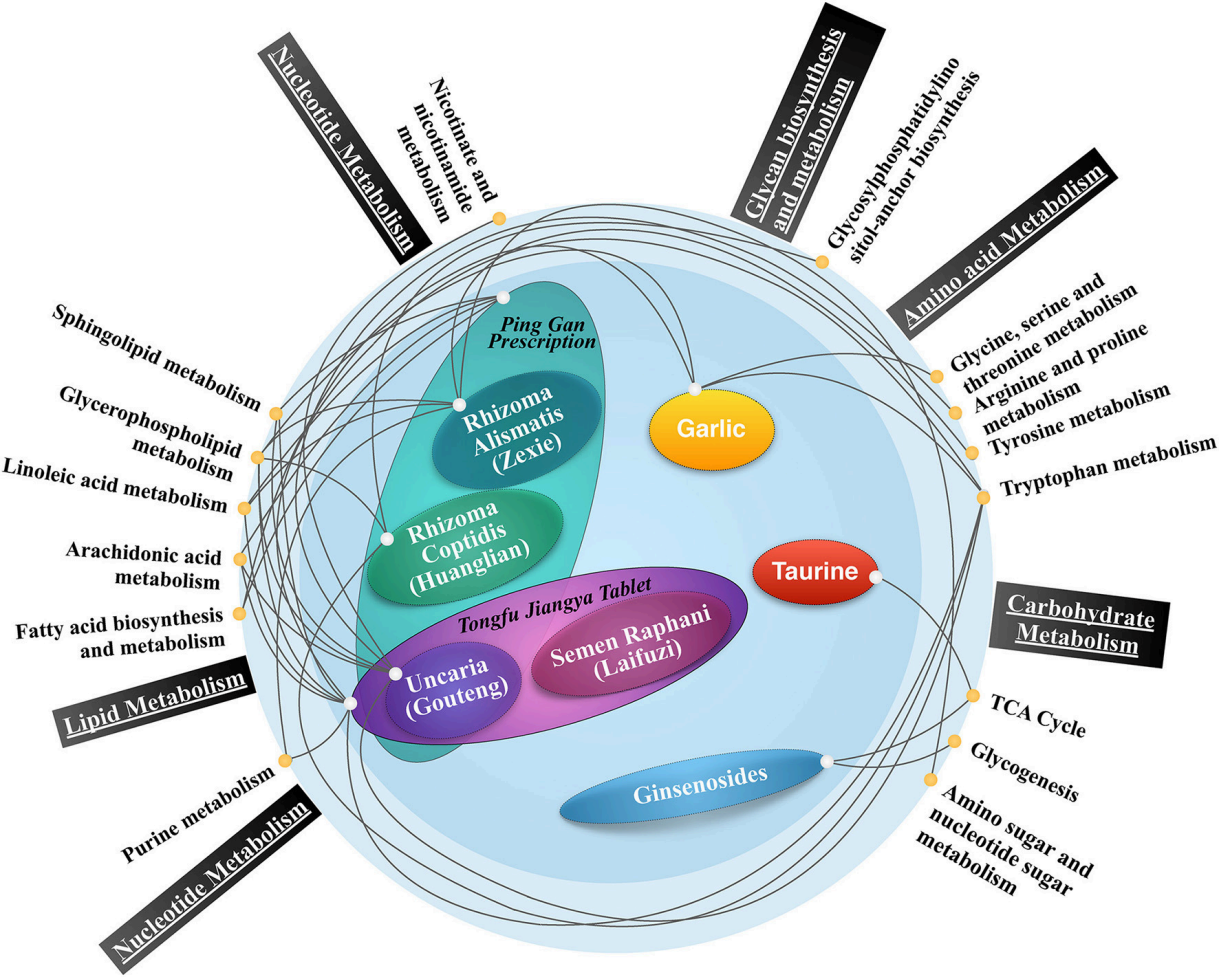
The antihypertensive herbal drug/formula- metabolic pathway map. Present metabolomics studies showed that antihypertensive herbal drugs/formulas are connected with carbohydrate metabolism, lipid metabolism, amino acid metabolism, and other major metabolic pathways, indicating the potential role of metabolic alternations induced by traditional Chinese medicine in treating hypertension. The interconnected network revealed that the two most prominent substance metabolisms activated by traditional Chinese medicine could be lipid and amino acid, as they have more frequent and concurrent biological information communications denoted by connecting lines.

Unlike unified western medicine treatments, TCM therapies are adjusted in individual levels based on TCM pattern differentiation for each patient. When commencing a TCM-related intervention, stage of hypertension, the severity of subsidiary symptoms and patient's lifestyle are determinant factors for the selection of appropriate therapy. According to the theories of TCM, hypertension can mainly be linked with four TCM patterns: (1) liver yang ascending due to kidney yin deficiency, (2) qi and blood deficiency, (3) kidney essence vacancy, and (4) phlegm stagnation in the middle energizer (Zhang, 1985). Correspondingly, TCM formulas that have been frequently used include *Tianma Gouteng* decoction (Wang et al., 2013a), *Guipi* Decoction (Li et al., 2016), *Zuogui* Pills, and *Banxia Baizhu Tianma* decoction (Xiong et al., 2012). There are a plethora of herbal species in those formulas, such as *Tianma* [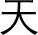

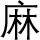
 *Gastrodiae Rhizoma (according to Chinese Pharmacopeia 2015)*, the dried tuber of *Gastrodia elata* Bl. (Orchidaceae)], *Gouteng* [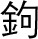

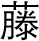
 *Uncariae Ramulus Cum Uncis*, stem of *Uncaria rhynchophylla* (Miq.) Miq. ex Havil. (Rubiaceae)], *Dihuang* [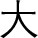

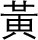
 *Rhei Radix et Rhizoma*, radix of *Rheum palmatum* L. (Polygonaceae)], *Banxia* [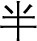

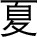
 *Pinelliae Rhizoma*, bulb of *Pinellia ternate* (Thunb.) Makino (Araceae)], *Baizhu* [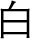

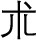
 *Atractylodis Macrocephalae Rhizoma*, rhizome of *Atractylodes macrocephala* Koidz. (Asteraceae)], *Huangqin* [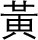

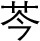
 *Scutellariae Radix, Scutellaria baicalensis* Georgi (Lamiaceae)], *Huanglian* [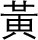

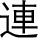
 *Coptidis Rhizoma*, rhizome of *Coptis chinensis* Franch (Ranunculaceae)], *Longdan* [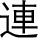

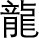
 *Gentianae Radix Et Rhizoma*, radix and rhizome of *Gentiana manshurica* Kitag. (Gentianaceae)], *Zexie* [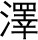

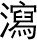
 *Alismatis Rhizoma*, bulb of *Alisma plantago-aquatica subsp. orientale* (Sam.) Sam. (Alismataceae)], *Juhua* [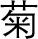

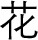
 *Chrysanthemi Flos*, flower of *Chrysanthemum morifolium* (Ramat.) Hemsl. (Asteraceae)], etc. (Zhang, 1985). A list of herbal plants commonly used in the clinical practice of Traditional Chinese Medicine for the treatment of hypertension is summarized in Table 1. Meanwhile, lifestyle modifications, such as diet and eating habit, emotion and physical exercise, are required to be taken in action by patients to effectively manage blood pressure. To probe the mechanism of how those multifaceted interventions work, many studies applied metabolomics approaches and yielded a large number of interesting discoveries. The experimental design and metabolomics outcomes including biomarkers, metabolic pathways were summarized in Table 2. An herbal drug/formula-metabolic pathway map was portrayed (Figure 1).

**Table 1:** List of plants used in the clinical practice of Traditional Chinese Medicine for the management of hypertension.

**CHN *Pinyin* name**	**ENG name (Pharmacopeia of china 2015)**	**CHN name**	**Latin name of original plant (Validated MPNS name)**	**Family name**	**Medicinal part**
*Tianma*	*Gastrodiae Rhizoma*	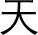 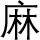	*Gastrodia elata* Blume	Orchidaceae	Tubers
*Gouteng*	*Uncariae Ramulus Cum Uncis*	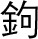 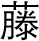	*Uncaria rhynchophylla* (Miq.) Miq. ex Havil.	Rubiaceae	Stem
*Dhuang*	*Rhei Radix et Rhizoma*	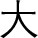 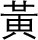	*Rheum palmatum* L.	Polygonaceae	Radix
*Banxia*	*Pinelliae Rhizoma*	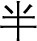 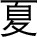	*Pinellia ternate* (Thunb.) Makino	Araceae	Bulb
*Baizhu*	*Atractylodis Macrocephalae Rhizoma*	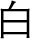 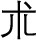	*Atractylodes macrocephala* Koidz.	Asteraceae	Rhizome
*Huangqin*	*Scutellariae Radix*	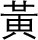 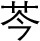	*Scutellaria baicalensis* Georgi	Lamiaceae	Radix
*Huanglian*	*Coptidis Rhizoma*	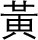 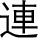	*Coptis chinensis* Franch	Ranunculaceae	Rhizome
*Longdan*	*Gentianae Radix Et Rhizoma*	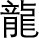 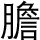	*Gentiana manshurica* Kitag.	Gentianaceae	Radix&Rhizome
*Zexie*	*Alismatis Rhizoma*	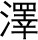 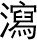	*Alisma plantago-aquatica subsp. orientale* (Sam.) Sam.	Alismataceae	Bulb
*Juhua*	*Chrysanthemi Flos*	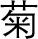 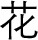	*Chrysanthemum morifolium* (Ramat.) Hemsl.	Asteraceae	Flower
*Laifuzi*	*Raphani Semen*	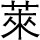 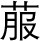 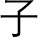	*Raphanus raphanistrum subsp. sativus* (L.) Domin	Brassicaceae	Seed
*Dasuan*	*Allii Sativi Bulbus*	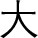 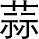	*Allium sativum* L.	Amaryllidaceae	Bulb

*MPNS, Medicinal Plant Name Service portal (http://mpns.kew.org/mpns-portal/), the Royal Botanic Gardens, Kew*.

**Table 2:** Characteristics of metabolomics studies of TCM in lowering blood pressure in animal models.

**Study ID**	**Treatment**	**Model**	**Control**	**Age**	**Sample**	**Platform**	**Potential antihypertensive biomarkers**	**Key pathways**
Aa et al., 2010	Total Ginsenosides, 30 or 3 mg/kg, ip daily for 8 weeks	SHR	Model and WKY rats	10–18 weeks	Plasma	GC-TOF/MS	**↑ metabolites:** Glycerol-2,3-Diphosphate, Glycerol-3-phosphate, Fumarate, ^1^H-Indole-3-propanoate, Glycerate, Ornithine;	Metabolism of lipids, the tricarboxylic acid cycle (TCA), glucose and amino acid turnover
							**↓ metabolites:** Oleic acid, Linoleic acid, Palmitic acid, 9-(Z)-Hexadecenoic acid, Arachidonic acid, alpha-Ketoglutarate, Citrate, Pyruvate, 3-Hydroxybutyrate, Aconitate, Tryptophan, Cystine, Glutamate, Cysteine, Creatinine, Glucose;	
Jiang et al., 2012a	Extract of Pinggan formula^*^, 18.336 g/kg, p.o. once daily for 15 days	SHR	Model, Captopril, and Wistar rats	Nil	Plasma	UPLC-QTOF-MS	**↑ metabolites:** PE(P-16:0e/0:0); 2-oxo-4-methylthio butanoic acid;	Sphingolipid metabolism, primary bile acid biosynthesis
							**↓ metabolites:** LysoPC(22:6), LysoPC(20:4), LysoPC(18:1), cholylglycine, SPP;	
Akira et al., 2013	Taurine aqueous solution (3%)in replacement of water, free access	SHR	Blank model	14 gfwk	24 h urine	^1^H NMR spectroscopy	**↑ metabolites:** Phenylacetylglycine, p-Cresol glucuronide, Isethionate, Hippurate, Acetate, Betaine, p-Cresol Sulfate;	TCA cycle
							**↓ metabolites:** α-Ketoglutarate, Citrate, Succinate;	
Jiang et al., 2015	Tengfu Jiangya tablet^*^, 100 mg/200 g, daily for 4 weeks	SHR	Model, Valsartan, and WKY rats	8 weeks	Serum	LC-TOF/MS	**↑ metabolites:** PE(22:2/15:0), L-Tryptophan, Citrulline, Gamma-Linolenic acid;	Sphingolipid metabolism, glycerophospholipid metabolism, tryptophan metabolism, arginine and proline metabolism, arachidonic acid metabolism, linoleic acid metabolism
							**↓ metabolites:** Sphinganine, Ceramide, LysoSM(d18:1), PC(20:5/15:0), LysoPC(16:0), L-Kynurenine, Xanthuiulrenic acid, L-Proline, Leukotriene D4;	
Chu et al., 2016	Rhizoma Alismatis extract, 10.001 g/kg, p.o., daily for 4 weeks	SHR	Model and Wistar rats	Nil	Serum	HPLC-TOF/MS	**↑ metabolites:** dGTP, 4-Hydroxyphenylacetylglutamine, Adenosine;	Glycerophospholipid metabolism, purine metabolism, linoleic acid metabolism, amino sugar and nucleotide sugar metabolism, tyrosine metabolism
							**↓ metabolites:** LysoPC(16:1), LysoPC(16:0), LysoPC(18:4), LysoPC(18:1), LysoPC(18:0), 12,13-EpOME, N-Acetylneuraminic acid, Deoxyguanosine, l-Octanoylcarnitine;	
Xie et al., 2016	Extract of Pinggan formula^*^, 18.336 g/kg, p.o. once daily for 15 days	SHR	Model and Wistar rats	Nil	Plasma	GC-MS	**↑ metabolites:** 9,12-Octadecadienoic acid, Octadecanedioic acid;	Fatty acid biosynthesis and metabolism, linoleic acid metabolism, arachidonic acid metabolism
							**↓ metabolites:** Hexadecanoic acid, Arachidonic acid, Elaidic acid ;	
Yang et al., 2017	Rhizoma Coptidis extract, 10.001 g/kg, igas daily for 4 weeks	SHR	Model and WKY rats	12 weeks	Serum	HPLC-TOF/MS	**↑ metabolites:** Fatty acid, Stearic acid, TX, PC [18:2 (9Z, 12Z)/16:0], Adrenic acid, PE [18:1 (9Z)/16:1 (9Z)];	Glycerophospholipids metabolism, tryptophan metabolism and glycosylphosphatidylinositol anchor biosynthesis
							**↓ metabolites:** 5-HTP, Sphingomyelin [d18:0/16:1 (9Z)], Docosapentaenoic acid and LysoPC (17:0)	
Matsutomo et al., 2017	S1PC (6.5 mg/kg) or SAC (7.9 mg/kg) 10 ml/kg, p.o., once daily for 10 weeks	SHR	Model and WKY rats	9 weeks	Plasma	LC-qOrbitrap/MS	**↑ metabolites:** Tryptophan, LysoPC(16:0), LysoPC(18:3), LysoPC(20:4);	Glycine, serine and threonine metabolism, tryptophan metabolism, glycerophospholipid metabolism, fatty acid biosynthesis
							**↓ metabolites:** Betaine, 3-(Acetyloxy)-2-Hydroxypropyl icosanoate, Caproic acid;	
Liu et al., 2018	Uncaria, 10.001 g/Kg, igas daily for 4 weeks	SHR	Model and Wistar rats	12 weeks	Serum	HPLC-TOF/MS	**↑ metabolites:** PC(16:1(9Z)/14:1(9Z)), Nicotinamide riboside;	Sphingolipid metabolism, glycerophospholipid metabolism, arachidonic acid metabolism, nicotinate and nicotinamide metabolism, tryptophan metabolism
							**↓ metabolites:** Dihydroceramide, Ceramide, LysoPC (22:5), Thromboxane A2, 5-HTP;	
Tian et al., 2018	Tengfu Jiangya tablet^*^, 100 mg/200 g, igas daily for 4 weeks	SHR	Model and WKY rats	Nil	Serum	UPLC-Q-exactive-MS and iTRAQ- LC-Q-exactive-MS	**↑ metabolites:** Sphinganine, 17a-Hydroxypregnenolone, Creatinine, L-Tryptophan, 9,12,13-TriHOME;	Sphingolipid metabolism, linoleic acid metabolism, arachidonic acid metabolism, steroid hormone biosynthesis, arginine and proline metabolism, tryptophan metabolism, glycerophospholipid metabolism, purine metabolism, tryptophan metabolism
							**↓ metabolites:** 13-OxoODE, 9-OxoODE, Sphinganine 1-phosphate, Thromboxane, Serotonin, PA(16:0/16:0), LysoPC (22:4), LysoPC (22:5), LysoPC (O-18:0), L-Arginine, 9(S)-HPODE, Linoleic acid, LPA (0:0/18:0), Xanthine, Phytosphingosine, 5,6-Dihydroxyprostaglandin F1a, LysoPC (22:6), 12(S)-HPETE;	

* *Ping Gan formula and Tengfu Jiangya tablet are Chinese medicine formulas used in studies exploring antihypertensive effect by metabolomics. According to literature, those expert opinion-based formulas have been widely used in the affiliated hospital of Shandong University of Chinese Medicine for many years*.

### Animal Studies in Herbal Medicine Treating Hypertension

#### Different TCM Treatment Principles, Similar Metabolic Response?

Herbal medicine is a major branch of traditional Chinese medicine, which has been used to treat cardiovascular diseases (CVDs) for over 2,000 years. Modern studies found that herbs used alone or in combination may elicit alterations of a wide spectrum of metabolites. For example, *Uncariae Ramulus Cum Uncis* is one of the most popular antihypertensive herbs in TCM. It is the key component of *Tianma Gouteng Yin*, which is a popular formula used for the treatment of hypertension of liver-yang ascending syndrome. Liu et al. applied HPLC-TOF/MS to characterize the *Uncariae Ramulus Cum Uncis*-induced metabolic alternations in the serum of SHRs (Liu et al., 2018). It showed that the SHR models have apparent metabolic perturbations as compared with normotensive rats. The irregularity of metabolism pattern of lipids (dihydroceramide, ceramide, PC, LysoPC, and TXA2), vitamin and amino acids (nicotinamide riboside, 5-HTP) is associated with hypertension. The antihypertensive effect of *Uncariae Ramulus Cum Uncis* is partially ascribed to the alterations of these metabolic pathways, including Sphingolipid metabolism, Glycerophospholipid metabolism, Arachidonic acid metabolism, Nicotinate, and nicotinamide metabolism, Tryptophan metabolism. Several relevant metabolites were considered biomarkers of the therapeutic effect of *Uncariae Ramulus Cum Uncis*, including PC [16:1(9Z)/14:1(9Z)], Nicotinamide riboside, Dihydroceramide, Ceramide, LysoPC (22:5), Thromboxane A2, 5-HTP.

Another study by Jiang et al. found *Tengfu Jiangya* Tablet (TJT) (Composition and application of the formula in Table 3), which is a Chinese patent compound constituted by *Uncariae Ramulus Cum Uncis* and *Raphanus raphanistrum subsp. sativus* (L.) Domin (*Laifuzi* 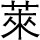

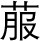

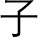
), may lower blood pressure through a similar mechanism (Jiang et al., 2015). Their study showed that in addition to the three abovementioned pathways affected by *Uncariae Ramulus Cum Uncis* alone, Arginine and proline metabolism and Linoleic acid metabolism are important to the effect of the *Uncariae Ramulus Cum Uncis*-containing compound. An in-depth study from Tian et al. integrated proteomics analysis into the metabolomics analysis and indicated that TJT improved blood pressure in SHRs via regulating three major pathways and their key catalyzing enzymes. The possible pathways are the kallikrein-kinin pathway, the lipid metabolism pathway, and the PPARγ signaling pathway (Tian et al., 2018). Biologically, those metabolic pathways are associated with impaired NO production, inflammation and vascular smooth muscle cells (VSMCs) apoptosis and prefiltration. Those studies highlighted that the TCM formula TJT may regulate lipids, vitamin and amino acids metabolism to improve NO production, inhibit inflammation and vascular remodeling, to further lower BP and exert cardiovascular protective effect.

**Table 3:** Composition and application of the antihypertensive formulas.

**Name**	**Herb name**	**Original plant (MPNS)**	**Medicinal part**	**Preparation**	**Dose**	**References**
*Tengfu Jiangya* tablet	*Gouteng* 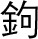 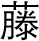	*Uncaria rhynchophylla* (Miq.) Miq. ex Havil.	Stem	Dried	Unreported	Jiang et al., 2015; Tian et al., 2018
	*Laifuzi* 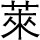 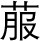 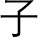	*Raphanus raphanistrum subsp. sativus* (L.) Domin	Seed	Dried	Unreported	
*Ping Gan* formula	*Huanglian* 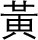 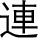	*Ranunculaceae Coptis chinensis* Franch	Rhizome	Dried	60 g	Jiang et al., 2012a
	*Gouteng* 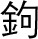 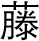	*Uncaria rhynchophylla* (Miq.) Miq. ex Havil.	Stem	Dried	150 g	
	*Zexie* 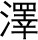 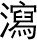	*Alisma plantago-aquatica subsp. orientale* (Sam.) Sam.	Bulb	Dried	60 g	
	*Luhui* 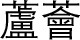	*Liliaceae Aloe Barbadensis* Miller	Leaf	Dried	5 g	

*MPNS, Medicinal Plant Name Service portal (http://mpns.kew.org/mpns-portal/), the Royal Botanic Gardens, Kew*.

As aforementioned, *Uncariae Ramulus Cum Uncis* is suitable for patients with liver Yang hyperactivity; while for hypertensive patients who present predominant symptoms of kidney Yin deficiency with water retention *Alismatis Rhizoma* and *Liuwei Dihuang Wan* are frequently prescribed in clinical settings. It is quite interesting that different herbs selected under different TCM treatment principles induced a similar metabolic reaction. For instance, Chu et al. showed that oral administration of *Alismatis Rhizoma* (which is a chief constituent of the *Liuwei Dihuang Wan*) alone in SHRs led to significant BP reduction; the antihypertensive effect was also related to the adjustment of glycerophospholipid metabolism and purine metabolism (Chu et al., 2016). Metabolic pathway analysis and biological property interpretation indicated that *Alismatis Rhizoma* may also exert hypotensive and cardiovascular protective effect by inhibiting endothelial cell apoptosis, VSMC proliferation and promoting NO production. The intermediate metabolites through which the herb might work include LysoPCs, dGTP, 4-Hydroxyphenylacetylglutamine, Adenosine, 12,13-EpOME, N-Acetylneuraminic acid, Deoxyguanosine, and Octanoylcarnitine. It is still unknown whether or not those pathways or metabolites represent a common mechanism of different treatments. To understand this could be helpful for us to explore new therapeutics of hypertension. More studies are needed to further address the possibly shared mechanism.

#### Different Compositions of Herbal Prescriptions, Similar Metabolic Response?

Moreover, *Coptidis Rhizoma*, or namely *Huang Lian* in Chinese, is widely used in TCM for eliminating CVD risk factors. Phytoconstituents of *Coptidis Rhizoma* include berberine, coptisine, palmatine, epiberberine, jatrorrhizine, and magnoflorine, all of which are potential cardiovascular protective agents. Through them, *Coptidis Rhizoma* exhibits anti-atherosclerotic effect, blood lipid lowering effect, anti-obesity effect and anti-hepatic steatosis effect (Tan et al., 2016). Though the cardioprotective effects of *Coptidis Rhizoma* are extensively investigated, the metabolic response to blood pressure regulation is rarely reported. Yang et al. performed an untargeted metabolomics analysis of the SHR serum samples to characterize the alternations of metabolic mode induced by *Coptidis Rhizoma*, to further elucidate its blood-lowering mechanism (Yang et al., 2017). It showed that 4-week gastric infusion of *Coptidis Rhizoma* reduced SBP of SHRs. In line with BP reduction, metabolomic profiles of SHRs were evidently altered toward that of normotensive rats. Ten endogenous metabolites were identified as potential biomarkers of the antihypertensive effect, including Fatty acid, Stearic acid, TX, PC, Adrenic acid, PE, 5-HTP, Sphingomyelin, Docosapentaenoic acid, and LysoPC. This study stated that *Coptidis Rhizoma* lowered blood pressure via initiating a metabolic network modulative effect, which involves the modulation of fatty acid biosynthesis and metabolism, tryptophan metabolism, and glycosylphosphatidylinositol anchor biosynthesis pathways.

TCM herbal formulas are usually made of at least three herbs. Therefore, they are considered more suitable to treat complicated conditions. *Pinggan* Prescription (PGP) is a *Coptidis Rhizoma*-enriched TCM herbal formula that is prescribed to hypertensive patients with internal heat syndrome (Composition and application of this formula in Table 3). Xie et al. found that PGP not only regulated fatty acid biosynthesis and metabolism but also significantly inhibited arachidonic acid (ArA) metabolism and augmented linoleic acid metabolism (Xie et al., 2016). Jiang et al. further compared the metabolic regulation effect of PGP with Captopril in lowing BP and found that PE and LysoPC are associated with blood pressure reduction. Moreover, a metabolite from the primary bile acid biosynthesis, Cholylglycine, is identified as a mediator of the antihypertensive effect of PGP, suggesting that PGP may regulate lipid metabolism through improving bile acid biosynthesis (Jiang et al., 2012a). From metabolomics analysis, it is quite interesting to point out that TCM formula with multiple herbs seems to have equivalent metabolic regulatory effect as a single herb. Are they identical in clinical effect? Or in improving metabolic dysfunction? What's the weight/significance of different pathways or mechanisms in lowering blood pressure? Does the concurrent pathways/metabolites shared by TCM formula and single herb take the full credits of blood pressure reduction? Or, does the minor difference in metabolic alternation help to reduce other CVD risks? Those are significant issues in this field worthy requiring further investigations in future.

#### Different Species of Herbal Materials, Similar Metabolic Response?

There are three major sources of TCM drugs including, herbal products, animal products, and mineral products (Koh and Woo, 2000). Herbal and animal products are commonly used for treating hypertension. Garlic, *Dasuan* [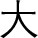

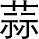
 *Allii Sativi Bulbus*, bulb of *Allium sativum* L. (Amaryllidaceae)] in Chinese, is both a necessary food ingredient and a TCM herb that can be used to manage intestinal disorders. The recent focus of research on the benefits of *Allium sativum* L. showed that aged garlic extract (AGE) is enriched with sulfur-containing amino acids such as *S*-1-propenylcysteine (S1PC) and *S*-allylcysteine (SAC) (Morihara et al., 2007; Matsutomo and Kodera, 2016). SAC has been demonstrated to cause BP reduction in renal hypertensive rats through an antioxidation mechanism (Cruz et al., 2007). *Matsutomo* et al. employed the LC-qOrbitrap-MS approach to characterize the regulatory effect of S1PC on the metabolic perturbation of SHRs (Matsutomo et al., 2017). Seven small-molecule metabolites were identified as markers of the antihypertensive effect initiated by S1PC, including Tryptophan, LysoPC(16:0), LysoPC(18:3), LysoPC(20:4), Betaine, 3-(Acetyloxy)-2-Hydroxypropyl icosanoate, Caproic acid. This study showed that AGE may exert the therapeutic effect on high BP through regulating glycine, serine, and threonine metabolism, tryptophan metabolism, glycerophospholipid metabolism and fatty acid biosynthesis.

One of the animal products, *Calculus Bovis* (*Niuhuang* in Chinese, which is dried cattle gallbladder stone) is a costly taurine-enriched animal product used by TCM to treat a variety of cardiovascular diseases and emergency conditions (Takahashi et al., 2009). Its major bioactive constituent, taurine, has been shown to have a distinctive therapeutic effect on cardiovascular system. Increased dietary intake of taurine lowered blood pressure in not only hypertension patients but also a group of hypertensive rodent models such as SHRs (Militante and Lombardini, 2002; Abebe and Mozaffari, 2011). Akira et al. quantified the urinary metabolites in taurine-treated SHRs with ^1^H NMR spectroscopic metabolomics (Akira et al., 2013). The excretion of three TCA cycle intermediates including citrate, α-ketoglutarate, and succinate, was suppressed in SHRs by chronic taurine intake. However, the output of phenylacetylglycine and p-cresol sulfate was increased. Taurine may exert hypotensive effect through two aspects, the acceleration of metabolic acidosis and the regulation of microflora metabolism. How metabolic acidosis accelerated by citrate reabsorption escalation in renal proximal tubules can be linked to BP reduction is hardly explained now. Taurine may fluctuate the gastrointestinal pH microenvironment to interfere the proliferation and metabolism of commensal microbes hosted in guts, such as Clostridium difficile, resulting in the increase in pheny-lacetylglycine and p-cresol sulfate (Clayton et al., 2009). The agitated microbial community may further increase the sulfonation reaction which converts taurine to inorganic sulfurs (Hepner et al., 1973). Another intermediate metabolite, succinyltaurine, which is structurally related to taurine, is a characteristic urinary compound of normotensive rats of at least 8-week old. While in SHRs of any age, the presence of such compound is undetectable. It is postulated that this metabolite is essential to the persistence of hypertension rather than the initiation of blood pressure elevation. A genetic diminish of enzymes and genes which are responsible for the formation of this metabolite in SHRs could be a probable reason for the persistent blood pressure elevation in the SHR model. This highlights the possible mechanism how supplementation of taurine rebalances the level of succinyltaurine and leads to BP reduction (Akira et al., 2010).

Those studies indicate that herbal product and animal product may lower blood pressure largely based on different metabolic regulation effect. Possibly, this is due to the distinction in effective constituents existed in different materials. Because the chemical compound of an animal product may share more chemical similarities with metabolites in the animal model or in human, it thus can be efficiently metabolized and utilized. However, compounds of an herbal product may experience more rounds of chemical or biological conversions before it enters certain metabolic pathways.

#### Metabolic Regulation: A Holistic Effect on all Clinical Outcomes?

BP reduction induced by TCM could be a holistic response coordinated by multiple mechanisms, due to the affluence of bioactive components that TCM herbs have and also their synergistic use. Metabolic regulation may not act independently to decrease BP. There is a good chance that other signaling pathways in parallel also produced substantial effects. For example, the renin-angiotensin system (RAS) represents a critical modulator of blood pressure homeostasis, the dysfunction of which is a common cause of the development of hypertension (Riet et al., 2015). TCM treatment can also reverse the abnormality of these systems. He et al. found that an *Coptis chinensis* Franch -constituted formula, *Yiqi Huaju* formula, lowered blood pressure and serum lipids level through inhibiting mRNA expression of the renal renin, angiotensin II (Ang II), and Ang II receptor, type 1 (AT1R), and inhibited the protein expression of renal AT1R and Ang II receptor type 2 in a high-salt and high-fat diet induced hypertension rat model (He et al., 2015).

Another study by Lee et al. found that another formula with *Coptidis Rhizoma, Jiawei Sanhuang Xiexin Tang* (HVC1), yielded a BP lowering effect in resemblance to that of Ca^2+^ channel blockers, which blocks the entry of extracellular Ca^2+^ via ligand-gated Ca^2+^ channels and voltage-gated Ca^2+^ channels (Lee et al., 2015). This study also showed that another possible mechanism of HVC1-caused vasorelaxation and BP reduction was through upregulating NO formation and the NO-cGMP pathway. Since Moreover, since TCM diagnosis is made on the basis of the holistic condition of a patient, is the TCM-induced restoration of the perturbed metabolic network a singular reaction leading to hypotension, or a comprehensive reaction that improves all metabolic parameters? Further studies should not limit themselves to only one clinical outcome. Instead, they should study the metabolic syndrome as a whole package of symptoms and include more metabolic indices to further build robust correlations between clinical improvement and metabolome restoration.

#### Metabolomics to Surveillance Drug-Herb Interactions and the Safety of TCM?

The abundant bioactive macro-/micro-molecules that herbal plants contain can bind to many generic pharmacological targets to generate therapeutic effects. The reaction may interfere with the pharmacokinetics or pharmacodynamics of conventional pharmacotherapies. The interplay between herb and drug may augment/ameliorate the pharmacological actions or toxicity of either component, which is recognized as herb-drug interactions (Fugh-Berman, 2000). Recent data indicate that the administration of herbs in combination with conventional antihypertensive agents causes serious consequences (Izzo et al., 2005). For instance, *Gingko Biloba* [
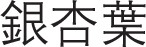
 *Ginkgo Folium*, leaf of *Ginkgo biloba* L. (Ginkgoaceae)] reduces the hypotensive effect of diuretic thiazide possibly through metabolic inhibition, and a 4-week treatment of *Gingko* significantly attenuates the antihypertensive effect of nicardipine which is metabolized by CYP3A2 in rats (Hu et al., 2005); *Mahuang* [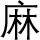

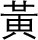
, stem of *Ephedra sinica* Stapf (Ephedraceae)] decreases the efficacy of beta-blockers and contributes to escalated BP when used together with monoamine oxidase inhibitors (MAOIs) (Sørensen, 2002). Other herbs such as *Allium sativum* L., *Gancao* [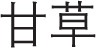 *Glycyrrhizae Radix et RhizomaGlycyrrhiza glabra*, radix, and rhizome of *Glycyrrhiza uralensis* Fisch. ex DC. (Fabaceae)], *Mugua* [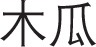 *Chaenomelis Fructus*, fruit of *Chaenomeles lagenaria* (Loisel.) Koidz. (Rosaceae)] may sabotage the effect of antihypertensive drugs. Inversely, herbs with no designated cardiovascular effect may yield a salient antihypertensive effect when administrated with modern drugs (Rosenkranz et al., 2012). Currently, the pharmaceutical constituent analysis is limited in pharmacosurveillance due to many herbal chemical compounds are undetectable or unknown.

Metabolomics that monitors the entire small-molecular events occurring at the metabolic level provides unique outlooks to observe the concurrent and independent actions of herbs and/or drugs. In fact, early metabolomics methods are applied in preclinical toxicity screening and ADMET (absorption, distribution, metabolism, excretion and toxicity) studies (Nicholson et al., 2002; Wishart, 2016). The Consortium for Metabonomic Toxicity has successfully utilized the ^1^H NMR spectroscopy to evaluate the xenobiotic toxicity in animal models (Lindon et al., 2007). Pharmacometabolomics is an increasingly attractive field that is yielding exciting and useful results regarding drug dosing and response measurements (Wishart, 2016). An excellent example is that in kidney transplantation rapid LC-MS-based metabolomics is used on a near-daily basis to monitor drug response and adverse events associated with multiple immunosuppressant combinational use (Shen et al., 2009; Brozmanová et al., 2010). Moreover, the metabolomics-based technological platform is the center of exposomics, which enables a top-down analysis of the exogenous and endogenous exposures to the internal “environment” of the human body (Rappaport, 2011). The exposomics analysis could be useful to identify potential drug interactions and monitor the residual of pesticide, heavy metal and other toxic contaminants within herbal plants. However, cautions are needed when using those technologies to detect the potential herb-drug interactions. A striking reason that hinders the use of such technologies is that certain methods still lack high sensitivity, such as the ^1^H NMR spectroscopy.

In hypertension treatment, a critical question whether TCM reduces blood pressure via mechanisms that differ from conventional medications so that the noxious herbal-drug interactions can hopefully be avoided. For instance, in TCM *Ginseng* [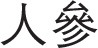 *Ginseng Radix et Rhizoma*, radix, and rhizome of *Panax ginseng* C.A.Mey. (Araliaceae)]is believed to have hypotensive actions, for the total ginsenosides (TG) it contains are capable of balancing the hypo- and hypertensive states (Yue et al., 2007). Aa et al. analyzed the plasma samples of SHRs treated with TG and different classes of conventional antihypertensive medications based on the GC-TOF/MS platform and further addressed the differential mechanism of TG as compared with conventional drugs (Aa et al., 2010). After 8-week treatment, the metabolic phenotype of SHRs exposed to TG significantly deviated from its originally aberrant metabolic phenotype and was recovered toward that of WKY rats (healthy state). The trajectory of metabolic profile alternation was in synchronization with the weekly blood pressure reduction. However, the metabolic perturbations in SHRs treated with captopril, amlodipine, terazosin, or hydrochlorothiazide didn't show sufficient correction toward normal metabolic profile, even though they all led to significant blood pressure reduction. Aa's study also found a connection between the regulatory effect of TG on lipid/sugar metabolism and the antihypertensive effect. Further analysis showed that TG restored the level of 51.8% metabolic biomarkers of hypertension to normal level, most of which were associated with lipid metabolism, TCA cycle and glucose-amino acid turnover. Conventional medications improved the level of no more than 29.6% hypertensive biomarkers. It can be inferred that the lipid metabolism-based antihypertensive effect is a core mechanism of TG as compared with conventional drugs.

### Investigating the Metabolic Response to TCM in Human Subjects

Using metabolomics to explore the mechanism of TCM in human subjects are still in infancy, even though their results can be implicational to clinical practice and drug discovery (Zhang et al., 2013a). Wu et al. and Wang et al. introduced metabolomics analysis into the research area of acupuncture specificity in treating functional dyspepsia and demonstrated that ^1^H NMR-based metabolomics platform could efficiently differentiate the metabolic response induced by several groups of acupoints and thus the actions of acupoint, demonstrating that metabolomics could be used to offer molecular perspectives for the solving of complex scientific questions in acupuncture therapy (Wu et al., 2010; Wang et al., 2012; Wu Q et al., 2016) Moreover, the abovementioned studies showed that various metabolites were triggered by acupuncture treatment in functional dyspepsia patients, including significant change of the levels of leucine/isoleucine, lactate, and glucose, and slight change of lipids level toward those of the healthy controls, which further revealed the putative mechanisms of acupuncture. However, metabolomics platform used in studies with human subjects that investigated the role of acupuncture in hypertension is still rare. The impedance mainly comes from clinical and technical concerns. Clinically, the efficacy of TCM herbs and non-pharmacological treatments such as acupuncture are still debatable (Lee et al., 2009; Wang and Xiong, 2013; Wang et al., 2013b). If metabolomics analysis is used to help assess the efficacy and address the possible mechanism, rigorous clinical research methodology like randomized controlled trial design should be employed in addition to biomedical analysis (Yuan and Lin, 2000). Valid control arms should be set in the trial for ruling out the possible non-specific/placebo effect (Paterson and Dieppe, 2005). The study by Yang et al. applied targeted metabolomics analysis to elucidate the metabolic response to acupuncture treatment in hypertensive patients (Yang et al., 2016). Oleic acid and myoinositol, were found most predictive not only to the blood pressure elevation but also to the therapeutic effect of acupuncture; the antihypertensive effect of acupuncture could be related to phosphatidylinositol signaling system and fatty acid biosynthesis. However, it is difficult to rule out the placebo-induced metabolic alternations in hypertensive patients due to lacking sham control, as studies showed that the antihypertensive effect of acupuncture could be partially, if not all, sourcing from placebo effect (Macklin et al., 2006). Similar pitfalls also exist in basic studies. One study by Wang et al. compared different stimulation parameters of acupuncture (with vs. without manual stimulation) in altering the urinary metabolome of spontaneous hypertensive rats and selected α-ketoglutaric acid, N-acetyl glutamic acid, and betaine as the candidate biomarker of manual acupuncture (Wang et al., 2017). Neither sham device nor non-acupoint was used in this study, leading to similar concerns regarding the role of placebo effect.

Further studies may consider using metabolomics analysis to differentiate the possible pathways through which true acupuncture and sham acupuncture lower blood pressure because the genuine effect of acupuncture on high blood pressure is a long-lasting debate (Kaplan, 2006; Moffet, 2007; Turnbull and Patel, 2007). Metabolomics studies by Gao et al., Liu et al., and Xu et al. used non-acupoint as a form of sham control to expel potential bias yielded by the non-specific factors in rat model of migraine and chronic atrophic gastritis model, respectively (Gao et al., 2014; Liu et al., 2017; Xu et al., 2017). Previous studies in major depressive disorders already demonstrated the capacity of metabolomics in separating the effect of antidepressant and placebo (Kaddurah-Daouk et al., 2013). Furthermore, to hypertension the maintenance of the therapeutic effect is critical. As blood pressure fluctuates and may lead to serious cardiovascular events in hypertensive patients, to explore whether acupuncture improves the stability of blood pressure is significant. Previous study by Zhang et al. in healthy subjects explicitly visualized the trajectory improvement of metabolic profile induced by acupuncture stimulation at “*Zusanli* (ST36)” point in 14 days (Zhang et al., 2016). Future study may also consider using metabolomics to probe the time-response curve of the antihypertensive effect of acupuncture.

Studies should also implement standardize interventional protocols. Pharmaceutical studies on TCM herbs should form generalized drug administration process and focus on the authentication and quality control of the herbal products by using geo-source verification and chemical biomarkers identification (Liang et al., 2004; Li et al., 2008). Procedural treatments, such as traditional herbal medicine and acupuncture, should form standard operating procedures (SOPs) to minimize interpatient and intergroup variations that may compromise the validity of clinical and metabolomics data (Zhao et al., 2009; Primrose et al., 2011). In order to secure the traditional values of treatment, TCM principles can be applied to guide the formation of semi-standardized or individualized intervention plans. This also requires an adequate sample size, because subgroup analysis on both clinical data and metabolomics data needs sufficient subjects to diminish variances and increase power. Feng et al. used ^1^H NMR-based metabolomics analysis to explore the mechanism of *Qingre Huatan* Decoction for treating phlegm-heat syndrome of hypertension in young male adults (Feng et al., 2015). The results showed that as compared with healthy controls the phlegm-heat pattern of hypertension in young adults can be metabolically characterized as the increase of Low-density lipoprotein/very low-density lipoprotein, N-acetyl glycoprotein, O-acetyl glycoprotein, and several lipids, and the decrease of High-density lipoprotein, Phosphatidylcholine, Lactate, Alanine, Choline/phosphocholine, Phosphorylcholine, Glucose, 3-Hydroxybutyrate, Citrate, and Glutamine in serum. The 4-week treatment restored the metabolic perturbations, revealing that *Qingre Huatan* Decoction regulates lipids, carbohydrate, and amino acid metabolism. The results have good internal validity because the study studied only one specific formula by focusing a peculiar syndrome of patients. However, to better address the needs of precision medicine, further studies are required to clarify the metabolic features of the different syndrome of hypertension and their TCM treatment with adequately powered design.

Many factors such as genetic variance (age and gender), disease history, psychological status, diet preferences, exercise, sleep pattern, and other lifestyle differences might lead to considerable variations of the metabolic mode within human body, as compared with animal models (Griffin et al., 2007; Johnson and Gonzalez, 2011). It is, therefore, critical for studies to control those aspects at the conception stage, as well as the data analysis and biological property elucidation stage. Preanalytical sample handling procedures (for no matter urine, serum, or other tissues) should be standardized (Bernini et al., 2011; Emwas et al., 2015). Moreover, metabolomics analysis is empowered to identify as many metabolites and metabolic pathway as possible and subsequently reveal the holistic/network effect of TCM herbal compound. One possible drawback is that the weight of each mechanism is unknown. To address this issue, omics and systems biology technology can be merged with other conventional methods to test the significance of the antihypertensive mechanisms (Jiang et al., 2012c; Lu et al., 2012).

## Further Perspectives in Metabolomics Studies of TCM Treating Hypertension

### Network Pharmacology to Discovery New Antihypertensives From TCM Treating Hypertension

Chinese herbal formulas may have significant regulatory effects on the metabolic perturbations in hypertension. However, the pharmacological property of each herb and the combining rules of herbs within TCM formula, known as “*Jun-Chen-Zuo-Shi*,” are still largely unknown. How to appreciate the systematic action and decipher the formula is still a challenge for TCM pharmacological studies, which is a key direction for future studies. TCM network pharmacology approaches proposed by Li et al can be used to seek holistic explanations for TCM formulas and thus can be a solution to the issues above (Li and Zhang, 2013). Network pharmacology employs omics technologies to detect different molecules (genes/enzymes/metabolites) and annotate them by comparing with specific databases (Wu et al., 2008; Yao et al., 2011). It can further pair those molecules to the clinical outcomes of diseases by using computing approaches including graph theory, statistical methods, data mining, modeling, and information visualization methods, which forms correlative patterns between network target and disease, where herb-target-disease associations can be developed. Furthermore, pharmaceutical ingredients can be detected by using analytical chemistry methods. By referring to certain TCM active ingredient database, the function of most ingredients can be annotated, which further contributes to the identification of active ingredients of herbal formula. For example, Zhang et al used network pharmacology to explore active components in Qing-Luo-Yin (QLY) formula for treating rheumatoid arthritis (RA) and identified kurarinone, matrine, sinomenine, berberine, and diosgenin from 235 ingredients of QLY to be anti-angiogenic and anti-inflammatory active ingredients (Zhang et al., 2013b). Moreover, pairing information of herbs based on the mutual effect in molecular and cellular level can be further explained based on computer algorithms such as the Distance-based Mutual Information Model (DMIM) (Li et al., 2010). Given the advantage of network pharmacology approaches in explaining the effect of the herbal formula, further studies may consider studying the network pharmacological effect of TCM by incorporating metabolomics with other omics methods and advanced computing science.

### Gut Microbiota-Host Interactions in TCM Treating Hypertension

TCM treatment-related biomarkers like hippurate, acetate, fumarate, pyruvate, and 3-hydroxybutyrate are endogenous metabolites co-metabolized by the host and its commensal gut microbiota. Studies in experimental models of hypertension confirmed that short-chain fatty acids (SCFAs), such as acetate and propionate, released by the fermentation of fiber by the gut microbiota are associated with lower blood pressure levels; there's also a growing body of evidence supporting the role of gut microbiota in the development and maintenance of high blood pressure state (Marques et al., 2018). Further studies demonstrated that SCFAs bind to metabolite-sensing G-Protein Coupled Receptors (GPCRs), such as GPR41, GPR43, GPCR olfactory receptor 51E2 (human)/Olfr78 (mice), to control gut hemostasis, host metabolism, and immune response (Pluznick et al., 2013; Natarajan et al., 2016; Marques et al., 2017). It is believed that immune response is a major path through which gut microbiota modulates human blood pressure, because those GPCRs are highly expressed in many subsets of T lymphocytes such as T helper 1, T_H_ 2, T_H_ 17, and regulatory T cells, all of which are involved in the regulation of BP and end-organ damage (Marques et al., 2018). Studies demonstrated that in the lung, the SCFAs downregulate pathways relating to cardiac fibrosis, cardiomyocyte apoptosis, cardiovascular system development and function. Moreover, in the heart and kidney, SCFAs such as acetate upregulate pathways related to circadian rhythm, which is an indicator of target organ damages associated with hypertension (Marques et al., 2018). Another critical aspect of gut microbiota's effect on host blood pressure regulation could be via autonomic nervous system-cardiorenal axis. Since TCM herbs are mainly metabolized and absorbed in the intestinal tract, the secondary metabolites may enter metabolic flux to regulate perturbations which are the main cause of hypertension. The overall collection of metabolites that were produced by gut microbiota are termed meta metabolome, for which metabolomics is also an essential tool to characterize the identity and property of the entirety (Turnbaugh et al., 2007; Wishart, 2007b). Therefore, studies may apply metabolomics approaches to monitor the meta metabolome of the gut microbiota and further validate the hypothesis in germ-free animal models and consider fecal transplantation a potential adjuvant to TCM antihypertensives.

### Metabolic Editing in TCM Treating Hypertension

**Figure 2:**
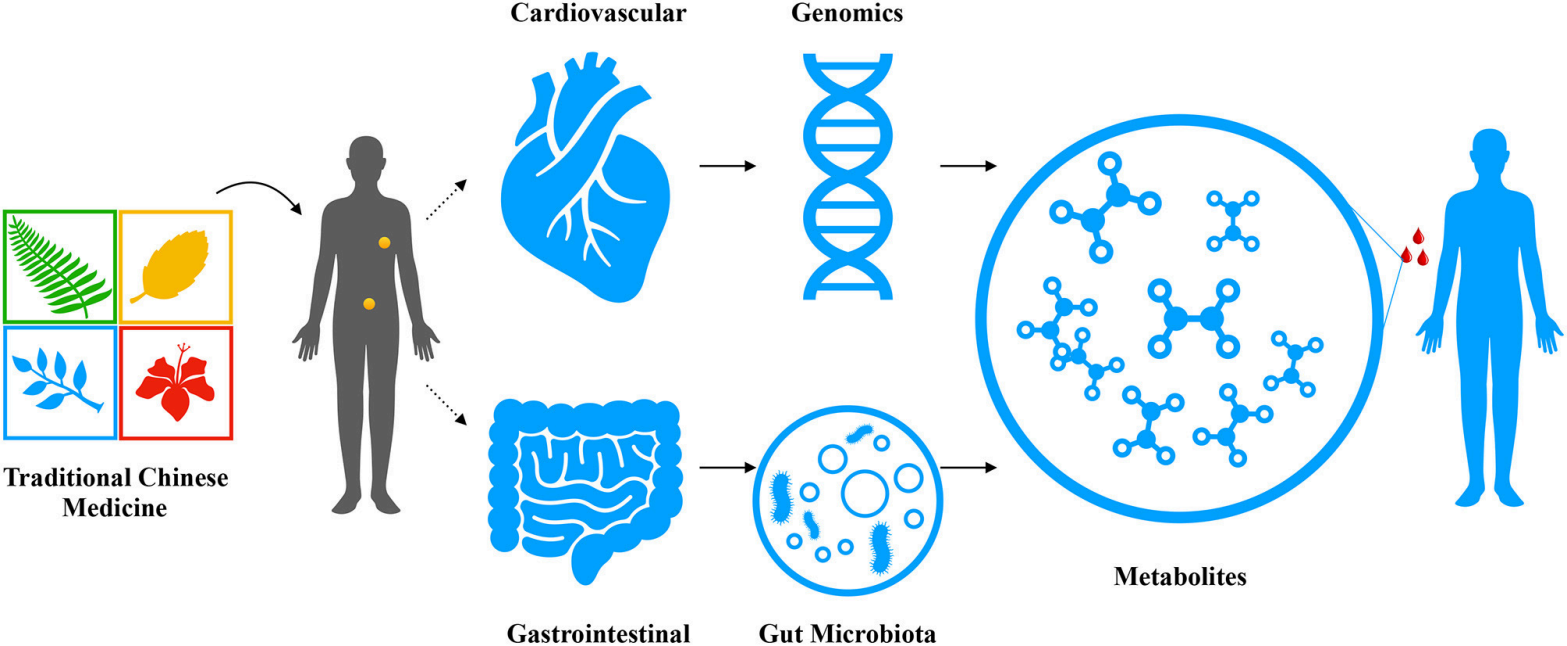
The antihypertensive herbal drug/formula- metabolic pathway map. Traditional Chinese medicine administrated by human for the purpose of treating hypertension and other cardiovascular diseases may take effect through two major paths: (1) though regulating genetic predispositions to module the enzymatic activities and consequently fluctuate the metabolome, which in turn changes the biological phenotype of a patient; (2) though rejuvenating the gut and restoring the intestinal hemostasis to reshape the composition and function of the gut microbiota, which will results in an increased production of blood pressure-benefit metabolites entering circulation to improve the clinical outcome.

Metabolism is editable. Human metabolism is regulated by both gene activities and environmental factors. Endogenously, it can be reprogrammed through gene editing; exogenously, it may be regulated by regulating human gut microbiota. Metabolic editing can be done by modifying diseases-related metabolic pathway genes instead of disease-specific genes. This method has been tested by Pankowicz et al. in hepatocytes for treating hereditary tyrosinemia (Pankowicz et al., 2016). Moreover, gut microbiota plays a significant role in regulating host metabolism as its metabolic products enter the host circulation system and participate in human metabolism. Therefore, gut microbiota provides access to edit human metabolism through altering environmental factors, such as through rebalancing intestinal pH, nutrients concentration, etc. As suggested by previous studies, TCM formulas have significant effects on certain metabolic pathways, which explains the blood pressure reduction induced by TCM. Here we propose the concept of metabolic editing induced by TCM to treat hypertension and other diseases. TCM may regulate metabolic disruptions in hypertension together by altering genetic dispositions and gut microbiota (Figure 2). However, it is unclear which part plays a major role in this process. Metabolic editing may offer new perspectives in understanding the effect of TCM treating hypertension and new strategies for treating hypertension. It helps to isolate hypertensive pathways that can be re-edited by TCM and other pharmaceutical agents to exert the hypotensive effect. Future studies may consider using herbal ingredients to edit affected metabolic pathways to reverse blood pressure escalation. Another access is via gut flora. As aforementioned, herbal medicine is mostly digested and absorbed in the digestive tract, the interplay between herbal pharmaceutical ingredients and gut microbiota is inevitable. Therefore, studies should first explore the mechanism of many herbs in regulating gut microbiota, particularly to find certain metabolic pathways that TCM alters through interacting with gut microbiota. Then, potential herbal ingredients can be screened to induce metabolic editing through gut flora. Metabolic editing may provide new visions and avenues for discovering antihypertensive and cardiovascular drugs from TCM.

## Conclusions

Metabolomics is used as a systems biology approach to explore the new pathophysiology of hypertension and elucidate the holistic molecular mechanisms of the action of TCM. Previous studies demonstrate that metabolomics helps to address the TCM syndrome differentiation rule for hypertension, which could be used as a novel diagnostic model for the optimization of hypertension management. Metabolic perturbations seen in hypertension population can be altered by TCM treatment, which is a crucial mechanism in the antihypertensive effect of TCM. TCM treatments, including herbal medicine and other non-pharmacological interventions, not only significantly rebalance the dynamics of metabolic fluxes, but also elicit a network-wide reorganization of metabolism. It has potentials to be used in combination with pharmacometabolomics and exposomics methods to monitor TCM drug safety and possible herb-drug interactions. This study also envisioned the applications of metabolomics in future TCM and hypertension studies, including (1) as a necessary component of network pharmacology approach to identify novel “network target and multiple components”; (2) to translate the meta metabolome into mechanistic insights of TCM modalities and further clarify the linkage between gut microbiota and host blood pressure regulation; (3) to measure the response of metabolic editing initiated by TCM treatments. Therefore, the emerging application of metabolomics in TCM treating hypertension denote great potentialities in identifying new hypertensive biomarkers and establishing novel antihypertensive therapeutics.

## Author Contributions

MY and LL conceived the idea of this review study. MY searched databases, collected literature, summarized the results and drafted the manuscript. LL supervised the whole process, provided suggestions for each step of the study, reviewed and edited the manuscript.

### Conflict of Interest Statement

The authors declare that the research was conducted in the absence of any commercial or financial relationships that could be construed as a potential conflict of interest.
